# Targeted delivery of nanomaterials with chemical cargoes in plants enabled by a biorecognition motif

**DOI:** 10.1038/s41467-020-15731-w

**Published:** 2020-04-27

**Authors:** Israel Santana, Honghong Wu, Peiguang Hu, Juan Pablo Giraldo

**Affiliations:** 1 Department of Botany and Plant Sciences, University of California, Riverside, CA 92521 USA; 2 Center for Plant Cell Biology, University of California, Riverside, CA 92521 USA; 3 Institute of Integrative Genome Biology, University of California, Riverside, CA 92521 USA; 4 Present Address: College of Plant Science & Technology, Huazhong Agricultural University, Wuhan, 430070 China

**Keywords:** Plant biotechnology, Plant biotechnology, Chloroplasts, Nanobiotechnology

## Abstract

Current approaches for nanomaterial delivery in plants are unable to target specific subcellular compartments with high precision, limiting our ability to engineer plant function. We demonstrate a nanoscale platform that targets and delivers nanomaterials with biochemicals to plant photosynthetic organelles (chloroplasts) using a guiding peptide recognition motif. Quantum dot (QD) fluorescence emission in a low background window allows confocal microscopy imaging and quantitative detection by elemental analysis in plant cells and organelles. QD functionalization with β-cyclodextrin molecular baskets enables loading and delivery of diverse chemicals, and nanoparticle coating with a rationally designed and conserved guiding peptide targets their delivery to chloroplasts. Peptide biorecognition provides high delivery efficiency and specificity of QD with chemical cargoes to chloroplasts in plant cells in vivo (74.6 ± 10.8%) and more specific tunable changes of chloroplast redox function than chemicals alone. Targeted delivery of nanomaterials with chemical cargoes guided by biorecognition motifs has a broad range of nanotechnology applications in plant biology and bioengineering, nanoparticle-plant interactions, and nano-enabled agriculture.

## Abstract (web summary)

Targeted delivery of nanomaterials to subcellular compartments could allow precision engineering of plant function. Here, Santana et al. show that quantum dots functionalized with a rationally-designed targeting peptide are preferentially delivered to chloroplasts where they can be used to tune organellar redox status.

## Introduction

The limited ability to target the delivery of biochemicals to specific plant tissues and organelles leads to inefficiencies of chemical inputs in agriculture and unintended alterations in plant function^1,2^. Only a fraction of agrochemicals, including nutrients and pesticides, reach the intended target in crops^1^ leading to environmental pollution^3^, low resource use efficiency in plants^4^, and inhibition of key plant physiological and developmental processes^5^. Although genetically modified organisms have proven to be of high value to understand plant function at the subcellular level, genome mutations or editing is accompanied by confounding effects including abnormal organ and tissue development^6^ and even leading to non-viable organisms^7^. Furthermore, the number of plants amenable for organelle genetic engineering is limited^8^. Nanomaterials are emerging as delivery vehicles for biomolecules in plants^9–13^ that can be tuned to control their translocation and distribution to plant cells and organelles.

Plant nanobiotechnology is a burgeoning field, which aims to develop and apply engineered nanomaterials (ENMs) for engineering and studying plant function^2,10,14–16^. Interfacing ENMs with plants is leading to significant advances towards addressing crucial challenges in plant genetic element delivery^11^, biochemical sensing^17–19^, and nutrient and pesticide delivery^20,21^. The broad potential for engineering plants utilizing ENMs with unique physical and chemical properties relies on circumventing plant barriers including cell walls and membranes, and improving the targeting to specific tissues and organelles^16^. Surface functionalization of ENMs with guiding moieties have enabled targeted biochemical delivery in non-plant eukaryotic cells^22^. Although the in vivo delivery efficiencies of nanoparticles coated with biorecognition ligands to target cancer cells are not higher than 2%^23^, localization in the nuclei of gold nanoparticles coated with SV40 large T antigen in HeLa cell cultures in vitro has been reported to be up to 60%^24^. Previous studies have reported the non-targeted delivery of DNA and biochemicals into plant cells and organelles by mesoporous silica nanoparticles through particle bombardment of leaf sections in vitro^9^ or by interfacing carbon nanotubes and their cargoes with isolated tobacco cells^25^. However, the destructive and invasive application of these approaches are not suitable for targeted delivery of nanoparticles to plant subcellular compartments in intact plants in vivo. Current approaches to improve in vivo nanoparticle delivery efficiency to specific plant cells or organelles are based on modifying nanoparticle properties such as size and charge but do not reach high levels of subcellular localization specificity^26^. For example, we have reported that negatively charged cerium oxide nanoparticles delivered into plant leaves have about 45% colocalization rates with chloroplasts^26^. However, most chloroplasts do not contain nanoparticles. Targeted nanomaterials guided by biorecognition ligands such as transit peptides have not been reported in plants to date because they cannot be directly translated from non-plant systems. Unlike mammalian cells, plant cells have a wall that acts as an additional barrier for nanoparticle translocation^27^. Nanoparticle uptake across plant cell walls is limited by the size of nanomaterials and cell wall pores^28^. Although the permeability of plant cell walls to nanomaterials has not been systematically characterized, it is expected to be dependent on plant species and nanoparticle properties including size and hydrophobicity^29^. For instance, amphiphilic nanoparticles (~40 nm) have been reported to translocate across leaf cells but not hydrophilic nanoparticles of similar or larger size. Furthermore, strong leaf background fluorescence from chloroplast pigments impairs our ability to easily track and colocalize nanomaterials in plants, thus requiring a specific design of fluorescent nanomaterials for both targeted and traceable biochemical delivery in plants in low fluorescent background windows.

**Fig. 1:**
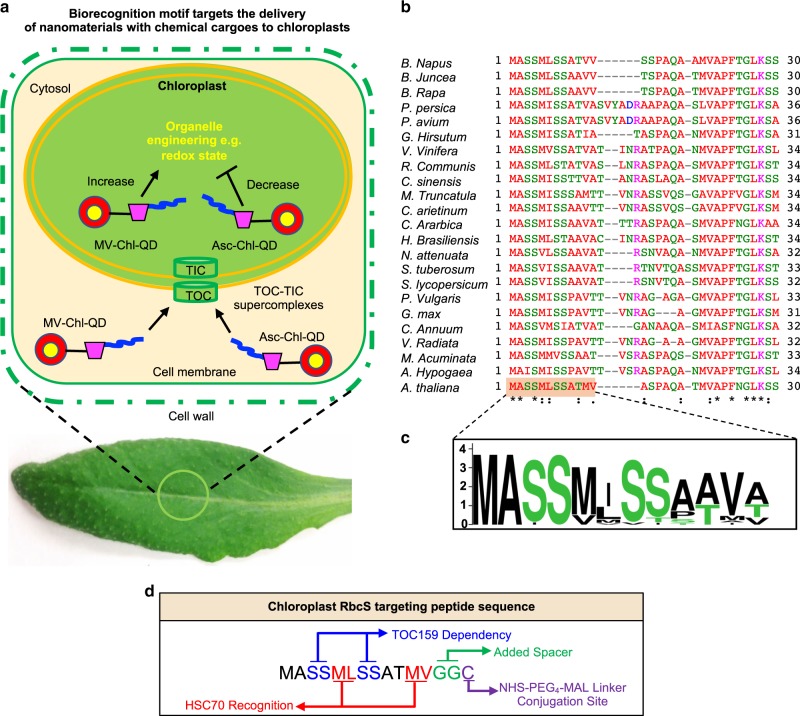
Targeted delivery of nanomaterials with chemical cargoes in plants enabled by a biorecognition motif. **a** Quantum dots coated with a chloroplast guiding peptide (in blue) and a β-cyclodextrin (β-CD) molecular basket (in magenta) enable loading of methyl viologen (MV-Chl-QD) or ascorbic acid (Asc-Chl-QD) and targeted modification of the redox status of chloroplasts in planta. The Rubisco small subunit (RbcS) targeting peptide is designed to bind to the translocon supercomplex on the chloroplast outer membrane (TOC). **b** Multiple sequence alignment analysis (Clustal Omega) of RbcS 1 A chloroplast transit peptide sequences in common dicot crops and *Arabidopsis thaliana* plants. Asterisk indicates the identical amino acids among all the aligned sequences. Colon and dot indicate conserved substitutions in which an amino acid is replaced by another one with similar properties. Empty space represents a non-conserved substitution. Dash lines are introduced for optimal alignment and maximum similarity between all compared sequences. **c** Frequency logo plot of RbcS 1 A targeting peptide consensus sequence across selected dicot species. A score of 4 on *y*-axis means 100% conserved. **d** Rational design of chloroplast guiding peptide based on RbcS peptide biorecognition motif for nanoparticle targeting and translocation across chloroplast membranes. The chloroplast targeting peptide includes recognition sites for chloroplast import machinery by TOC, a cysteine residue at the C-terminus for conjugation with NHS-PEG_4_-MAL linker, and two glycine (G) amino acids as spacers and for increasing the peptide solubility.

Chloroplasts are key organelles for plant bioengineering. These semi-autonomous organelles are essential for plant photosynthesis, act as signaling organelles, and play important roles in metabolite synthesis^30,31^. Thus chloroplasts are important for improving crop growth, stress tolerance, biopharmaceutical production, and developing synthetic biology tools^31^. Manipulation of chloroplast function through biomolecule or chemical delivery in plants is crucial for understanding the role of these plastids in plant biology and developing approaches for chloroplast bioengineering. However, chloroplast studies are limited to the need of targeted delivery platforms for specifically engineering plant organelle function in vivo. Instead, chloroplast research mainly relies on the generation of transgenic or mutant plants with altered development and function in a handful of model species amenable for genetic transformation^8^. Herein, we demonstrate approaches for designing novel ENM platforms guided by a peptide recognition motif that targets the delivery of chemicals to chloroplasts in wild-type *Arabidopsis thaliana* (Col-0) plants. Using these nanoscale platforms, we enabled the capability to specifically manipulate chloroplast function and redox status in vivo (Fig. 1a).

This study highlights approaches to design and synthesize novel ENM platforms that bypass biological barriers in plants such as cell walls, membranes, and organelle envelopes for in vivo traceable and targeted delivery of biochemicals to chloroplasts using guiding peptide recognition motifs. To demonstrate this targeted nanoparticle delivery and tracking approach in plants, we designed hydrophilic quantum dots (QD) coated with β-cyclodextrin molecular baskets with size that facilitates translocation through leaf cell wall pores, RbcS guiding peptide to recognize organelle membranes, high zeta potential to translocate across lipid bilayers, and optimal fluorescence emission range avoiding leaf background for in vivo imaging. The QD intrinsic fluorescence and heavy element composition (cadmium and tellurium) allows their specific imaging in plants by high spatial resolution confocal fluorescence microscopy and quantitative detection through advanced analytical tools including elemental analysis by ion coupled plasma mass spectrometry (ICP-MS).

## Results

### Quantum dots with rationally designed guiding peptide

Multifunctional fluorescent QD act as traceable chemical delivery platforms by forming inclusion complexes with chemicals such as methyl viologen (MV) and ascorbic acid (Asc) on their surface through conjugated β-cyclodextrins (β-CD) molecular baskets (Supplementary Fig. 1). The delivery of MV and Asc by QD allows tunable changes in chloroplast redox status by inducing or reducing superoxide anion production in this organelle with high specificity (Fig. 1a). Manipulation of chloroplast redox status has been associated with wide genetic and physiological responses in plants^32^. A conserved chloroplast targeting peptide, rationally designed from Rubisco small subunit 1 A (RbcS, genbank: OAP15425), was used to functionalize fluorescent QD for targeting chloroplasts in intact leaves of plants in vivo (Fig. 1b–d). To our knowledge, this is the first time a nanoparticle has been guided to a specific subcellular compartment in plants (e.g., chloroplasts) by mimicking the biorecognition mechanisms used for protein precursor delivery. An alignment of the peptide amino acid sequence with RbcS-peptide analogs from multiple dicotyledonous plant species indicates a high degree of conservation in its composition and sequence across crop and model plants (Fig. 1b, c). The RbcS transit peptide enables cytosolic recognition of proteins destined for import into plastids by the chloroplast outer membrane translocon TOC159 (Fig. 1a)^33^. TOCs recognize the N-binding domain of most pre-proteins destined to plastids and function in coordination with translocons at the inner membrane of chloroplasts (TICs) to allow import of pre-proteins into the chloroplast stroma^34^. Our ENM targeting sequence was truncated to the first 14 amino acids to minimize the increase in hydrodynamic diameter of functionalized QD and improve the penetration through leaf cell wall pores (Fig. 1d)^27^. A short sequence containing GGC was added to the C-terminal of the peptide as spacer. The terminal cysteine residue was further utilized as conjugation site to bind with a NHS-PEG_4_-MAL (succinimidyl-[(N-maleimidopropionamido)-tetraethyleneglycol] ester) linker onto the QD (Fig. 1d). The first 14 amino acids from the RbcS peptide sequence used to guide nanoparticles are highly conserved among dicots and contain functional biorecognition motifs allowing translocation across the chloroplast double membranes^33,35^. Thus, this targeted nanoparticle approach using RbcS-peptides is likely to have broad applicability in dicot plant species.

**Fig. 2:**
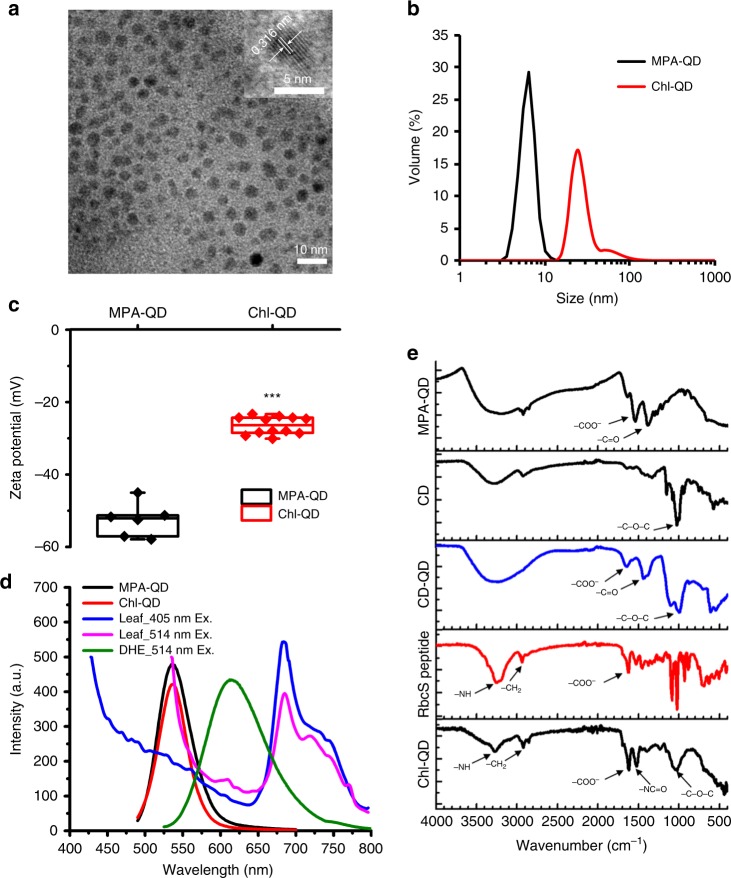
Design and characterization of multifunctional quantum dots with chloroplast guiding peptide. **a** Representative transmission electron microscopy image of MPA-QD showing the average diameter of the QD nanoparticle core of 4.3 ± 0.2 nm (*n* = 3). **b** Hydrodynamic diameter measured by DLS (pH 7) of MPA-QD (6.1 ± 0.5 nm, *n* = 4) and Chl-QD (24.5 ± 2.5 nm, *n* = 5). Values are means and ± indicates standard deviation. **c** High zeta potential of MPA-QD (−52.6 ± 4.7 mV, *n* = 6) and Chl-QD (−28.4 ± 3.8 mV, *n* = 17) that allows penetration through lipid bilayers in the cell membrane and chloroplasts. Box plot error bars represent standard deviation, boxes are the interquartile range from the first to the third quartile, and horizontal line represents the mean. Statistical comparison was performed by independent samples *t*-test (two tailed). *** indicates *P* < 0.001. **d** Fluorescence emission spectra of MPA-QD and Chl-QD in the range of low background fluorescence emission from leaves. **e** FTIR spectra of MPA-QD, β-cyclodextrin (β-CD) coated QD (CD-QD) and Chl-QD indicating successful functionalization of QD with β-CD and guiding peptide.

QD with β-cyclodextrin molecular baskets conjugated with targeting peptides (Chl-QD) allow the targeted delivery of biochemical cargoes into chloroplasts. The β-CD molecular basket composed of seven cyclic oligosaccharides enables “host-guest” formation with ascorbic acid or methyl viologen^36,37^ and can form inclusion complexes with a broad range of biomolecules and chemicals including metabolic intermediates (β-carotenes), or herbicides (MCPA and norflurazon) (Supplementary Table 1). At the terminal amine group located on the β-CD, a succinimidyl-dPEG-maleimide linker (NHS-PEG_4_-MAL) was added providing a selective conjugation site for cysteine residues located on the transit peptide. The QD core size of 4.3 ± 0.2 nm (± indicates standard deviation, *n* = 3) measured by transmission electron microscopy (TEM) (Fig. 2a), and the Chl-QD average hydrodynamic diameter of 24.5 ± 2.5 nm (± indicates standard deviation, *n* = 5) measured by dynamic light scattering (DI water, pH 7) (Fig. 2b) are under the maximum size for nanomaterials reported to translocate across leaf cell walls^27,29^. The synthesized Chl-QD are negatively charged with a zeta potential of −28.4 ± 3.8 mV (± indicates standard deviation, *n* = 17) in DI water (pH 7) (Fig. 2c) to facilitate translocation across plant lipid membranes. Nanoparticles with high zeta potential have been reported to passively penetrate through chloroplast envelopes and plant cell membranes^12,17,38,39^. Furthermore, QD have a high and stable fluorescence enabling in vivo tracking within plant tissues and cellular compartments^40^. The Chl-QD fluorescence peak was tuned to 540 nm to reduce interference with leaf background and fluorescent dyes used in this study (Fig. 2d). The QD exhibited a characteristic absorption peak at 465 nm in the UV-vis absorption spectrum (Supplementary Fig. 2a). In Fourier transmittance infrared spectra (FTIR) (Fig. 2e), significant characteristic bands for asymmetric glycosidic vibration (C–O–C) denoting β-cyclodextrin were detected at 1058 cm^−1^, and bands typical of type I and II amide bonds at 1615 and 1515 cm^−1^ supported successful conjugation of β-cyclodextrin and RbcS peptide on QD surface forming Chl-QD complex.

### Biorecognition targeted delivery of nanoparticles in vivo

**Fig. 3:**
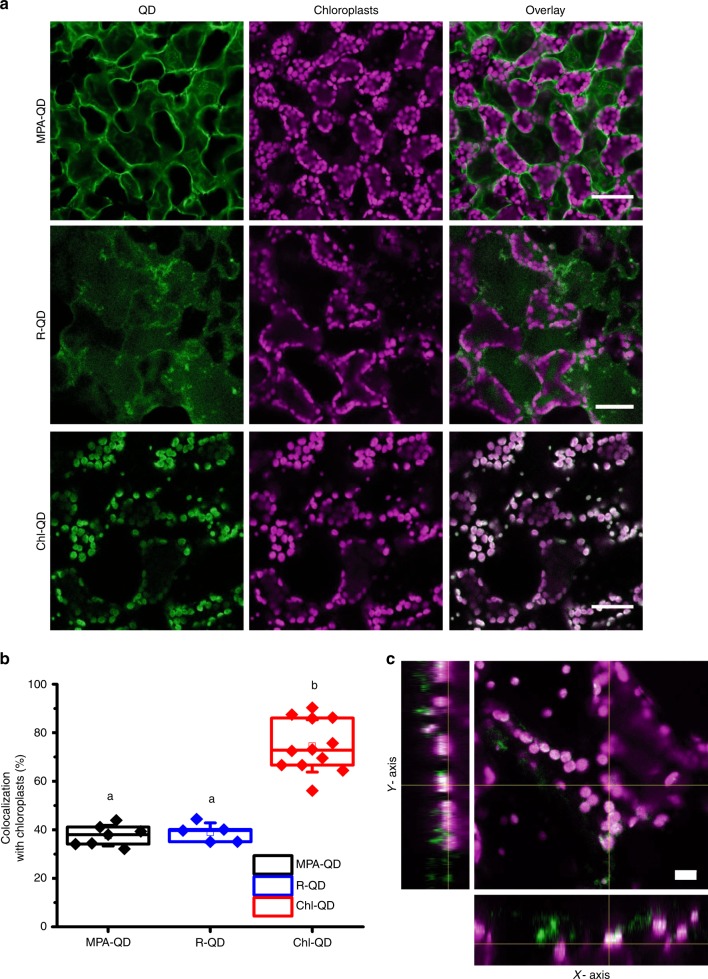
Targeted delivery of quantum dots to chloroplasts of Arabidopsis leaf mesophyll cells. **a** Confocal microscopy images of chloroplasts in leaf mesophyll cells indicating a higher degree of colocalization of QD coated with guiding peptide (Chl-QD, *n* = 12) with chloroplasts compared to QD without targeting peptide (MPA-QD, *n* = 7) and QD coated with a randomized amino acid sequence of the guiding peptide (R-QD, *n* = 5). Scale bar, 40 µm. **b** Colocalization rates of Chl-QD (*n* = 12) with chloroplasts compared to MPA-QD (*n* = 7) and R-QD (*n* = 5). Statistical comparisons were performed by one-way ANOVA based on Duncan’s multiple range test (two tailed). Lower case letters represent significance at *P* < 0.05. Box plot error bars represent standard deviation, boxes are the interquartile range from the first to the third quartile with squares as the medians, and horizontal line represents the mean. **c** Orthogonal views of different planes from confocal images (z-stack) of Chl-QD colocalization within chloroplasts. Scale bar, 10 µm.

The localization between Chl-QD and chloroplasts in leaf mesophyll cells of *Arabidopsis* plants was assessed by confocal fluorescence microscopy. QD lacking the targeting peptide (MPA-QD) and QD functionalized with a randomized RbcS sequence (R-QD, ASLSSMMATSGVGMC) were tested to validate the role of the conjugated chloroplast targeting peptide sequence on the Chl-QD localization in plant cells. We found similar chloroplast colocalization rates between MPA-QD (37.6 ± 4.2%) (± indicates standard deviation, *n* = 7) and R-QD (38.9 ± 3.9%) (± indicates standard deviation, *n* = 5) (Fig. 3a, b). In contrast, we observed two times higher percentage of chloroplasts containing Chl-QD coated with the guiding peptide (74.6 ± 10.8%) (± indicates standard deviation, *n* = 12) (Fig. 3a, b). No QD fluorescence was detected in buffer treated plants (Supplementary Fig. 3). The spatial distribution of QD within chloroplasts was visualized by orthogonal views, which are constructed from multiple Z-stack images (Fig. 3c, Supplementary Fig. 4, Supplementary Movie 1) collected at 2 μm per scanning layer, which is smaller than *Arabidopsis* chloroplast size (5–10 μm)^26^. These results demonstrate that our truncated RbcS-peptide guided the QD to chloroplasts with high targeted nanoparticle delivery efficiency and specificity in plants. Interestingly, the zeta potential of Chl-QD is significantly lower than MPA-QD (*P* < 0.001) (Fig. 2c) indicating that models based on increased nanoparticle charge for promoting chloroplast delivery^12,38,39^ are not sufficient to predict plant organelle localization of nanomaterials guided by biomolecule recognition motifs in plants in vivo.

**Fig. 4:**
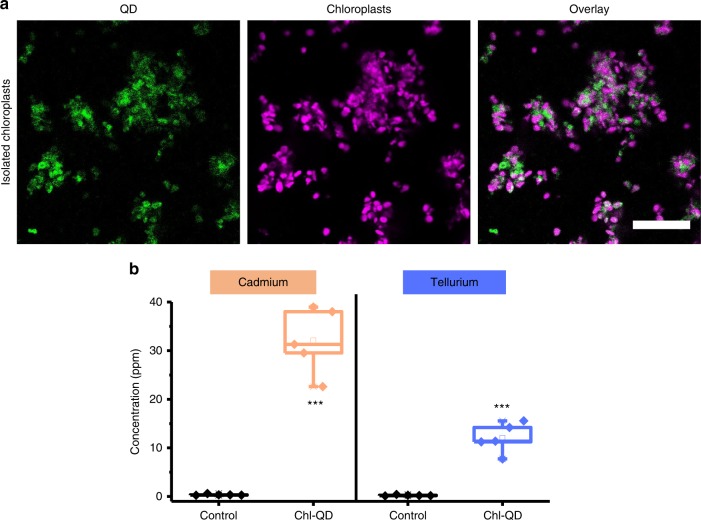
Detection of quantum dots in isolated chloroplasts by confocal microscopy and inductively coupled plasma mass spectrometry (ICP-MS). **a** Confocal fluorescence microscopy images of isolated chloroplasts that were targeted in vivo with CdTe quantum dots functionalized with a chloroplast guiding peptide (Chl-QD). **b** ICP-MS elemental analysis of cadmium and tellurium in isolated chloroplasts from plant leaves exposed to Chl-QD (*n* = 5) and controls (*n* = 5) infiltrated with TES buffer. Scale bar, 50 μm. Box plot error bars represent standard deviation, boxes are the interquartile range from the first to the third quartile with squares as the medians, and horizontal line represents the mean. Statistical comparison was performed by independent samples *t*-test (two tailed). *** indicates *P* < 0.001.

The presence of QD core elements, cadmium (Cd) and tellurium (Te), was confirmed by ICP-MS of isolated chloroplasts from leaves treated with Chl-QD and controls. Chloroplasts from *Arabidopsis* plants treated in vivo with Chl-QD (500 nM) or 10 mM TES buffer (pH 7.0) controls were isolated and imaged by confocal microscopy to record QD fluorescence (Fig. 4a). The concentration of Cd and Te measured by ICP-MS in Chl-QD treated samples was 32.08 ± 6.71 ppm (± indicates standard deviation, *n* = 5) and 12.02 ± 3.03 ppm (± indicates standard deviation, *n* = 5), respectively (Fig. 4b). In contrast, controls contained negligible amounts of Cd and Te 0.23 ± 0.12 and 0.36 ± 0.14 ppm (± indicates standard deviation, *n* = 5), respectively. Together, confocal microscopy and ICP-MS analysis demonstrate that nanomaterials coated with chloroplast transit peptide motifs (Chl-QD) translocate in leaf mesophyll cells and localize within chloroplasts (Fig. 3, and Fig. 4). The Chl-QD (200 nM) were also biocompatible in *Arabidopsis* leaves in which we did not observe toxicity effects in mesophyll cells up to 24 h measured by staining the nuclei of dead cells with PI (propidium iodide) dye (*n* = 4) (Supplementary Fig. 5).

### Chloroplast-targeted tuning of oxidative status

**Fig. 5:**
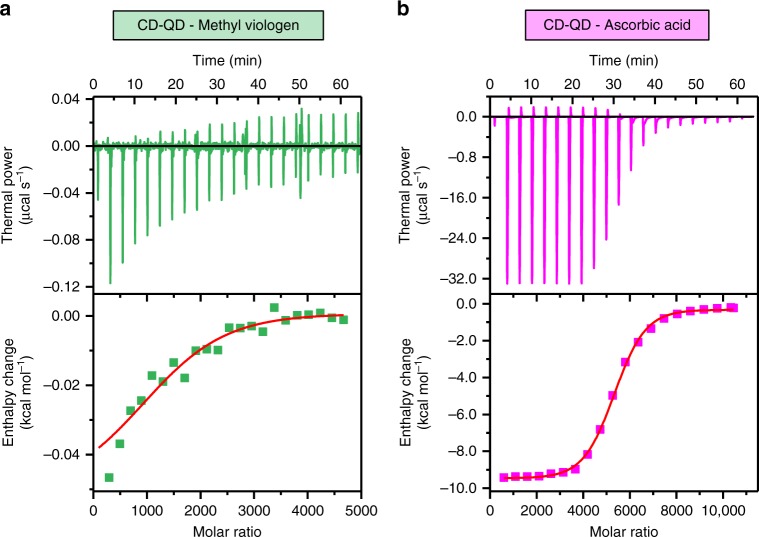
Isothermal titration calorimetry (ITC) of β-cyclodextrin coated quantum dots (CD-QD) with chemical cargoes. Thermograms (top) showing the thermal power peaks corresponding to each chemical injection and binding isotherms (bottom) representing the enthalpy change determined by integrating thermal power profiles for CD-QD interacted with **a** methyl viologen and **b** ascorbic acid. The ITC results indicate that CD-QD exhibit a higher binding affinity to both methyl viologen and ascorbic acid than MPA-QD. Squares represent raw data and line represents best-fit curves using a one-set-of-sites binding model.

As a proof of concept, we demonstrate that targeted biochemical delivery to chloroplasts using Chl-QD allows tunable changes in the redox status of these organelles. Chloroplast function is intrinsically related to the generation of reactive oxygen species (ROS)^41^. ROS play a dual role in plants as signaling or damaging molecules^42^. ROS accumulation in chloroplasts leads to declines in photosynthesis, plant growth and yield^43^. However, understanding the role of ROS in chloroplasts has been limited to research in plant model species amenable for genetic engineering and in mutants that often suffer from impaired growth and development^6,7^. To enable the manipulation of ROS levels in chloroplasts of wild-type plants in vivo, Chl-QD were loaded either with methyl viologen (MV) to generate superoxide anion in chloroplasts^44^ or ascorbic acid (Asc), a known scavenger of superoxide anion^45^. The absorption spectra of MV-Chl-QD and Asc-Chl-QD (Supplementary Fig. 2b) indicate the initial loading (60 μM) of MV or Asc in Chl-QD solution (Supplementary Fig. 2c, d). In addition, we assessed the binding between cyclodextrin coated QD (CD-QD) and MPA-QD to Asc or MV by isothermal titration calorimetry (ITC) (Fig. 5a, b, Supplementary Fig. 6a, b). ITC experimental data and best-fit binding curves of MV and Asc with CD-QD (Fig. 5a, b) provided the stoichiometry for calculating number of binding sites (*N*), association constants (*K*_*a*_), dissociation constants (*K*_*d*_), enthalpy (Δ*H*), and entropy (Δ*S*) changes (Table 1). The ITC analysis yielded that CD-QD have higher binding affinity to both Asc and MV than MPA-QD as indicated by lower dissociation constants or more binding sites per particle (Table 1, Supplementary Fig. 6). The *K*_*d*_ of 3.98 × 10^−5^ M for CD-QD and Asc is lower than that for MPA-QD and Asc (*K*_*d*_ = 5.52 × 10^−5^ M) and the number of binding sites on CD-QD for Asc (5130) is 10 times higher than MPA-QD. Although the *K*_*d*_ for CD-QD and MV (4.76 × 10^−5^ M) is similar to that for MPA-QD and MV (*K*_*d*_ = 4.81 × 10^−5^ M), the number of binding sites on CD-QD of 1580 for MV is more than 3 times higher than MPA-QD. The lower dissociation constant of Asc (*K*_*d*_ = 3.98 × 10^−5^ M) to CD-QD than MV (*K*_*d*_ = 4.76 × 10^−5^ M) is likely due to the high –OH group association between Asc and the cyclodextrin rim^36^. Furthermore, the *K*_*d*_ values for CD-QD are also an order of magnitude lower than that for β-CD reported by previous studies (10^−4^–10^−3^ M)^36,46,47^ indicating a higher binding affinity of CD-QD than β-CD to Asc and MV. The increase in the number of binding sites for Asc and MV on CD-QD reflects the ability of cyclodextrin molecules on these particles to act as molecular baskets for loading and delivery of biomolecules. As a result of either lower dissociation constant or more binding sites of CD-QD to Asc and MV (Table 1), the calculated fraction of bound Asc (96.05%) and MV (84.78%) onto the CD-QD was significantly increased relative to that of MPA-QD lacking cyclodextrins (Asc 54.94%, MV 56.73%) (Table 2). This increase of nanoparticle inclusion complexes with chemicals such as viologens using cyclodextrins^46^ enables the loading and subsequent release of chemicals to intended targets in plants.

**Table 1:** Isothermal titration calorimetry (ITC) thermodynamic parameters.

**Nanomaterials**	**Ligand**	***N*** **(sites)**	***K***_***a***_ **(M**^**−1**^**)**	***K***_***d***_ **(M)**	**∆*H*** **(cal mol**^**−1**^**)**	**∆*S*** **(cal mol**^**−1**^ **K**^**−1**^**)**
CD-QD	MV	1580	2.10 × 10^4^	4.76 × 10^−5^	−50.76	19.6
CD-QD	Asc	5130	2.51 × 10^4^	3.98 × 10^−5^	−9780	−12.7
MPA-QD	MV	458	2.08 × 10^4^	4.81 × 10^−5^	−594	17.7
MPA-QD	Asc	532	1.81 × 10^4^	5.52 × 10^−5^	−1693	13.7

Thermodynamic parameters including number of binding sites (*N*), association constant (*Ka*), dissociation constant (*Kd*), enthalpy change (*∆H*), and entropy change (*∆S*) of interactions between QD (CD-QD or control MPA-QD) and chemical cargoes (methyl viologen or ascorbic acid).

**Table 2:** Calculated bound and unbound fractions of chemicals on quantum dots.

**QD**	**Ligand**	**Bound fraction (%)**	**Unbound fraction (%)**
CD-QD	MV	84.78	15.22
CD-QD	ASC	96.05	3.95
MPA-QD	MV	54.94	45.06
MPA-QD	ASC	56.73	43.27

The calculated bound and unbound fractions of methyl viologen and ascorbic acid on QD using isothermal titration calorimetry analysis and concentrations of chemicals in nanomaterial solutions.

**Fig. 6:**
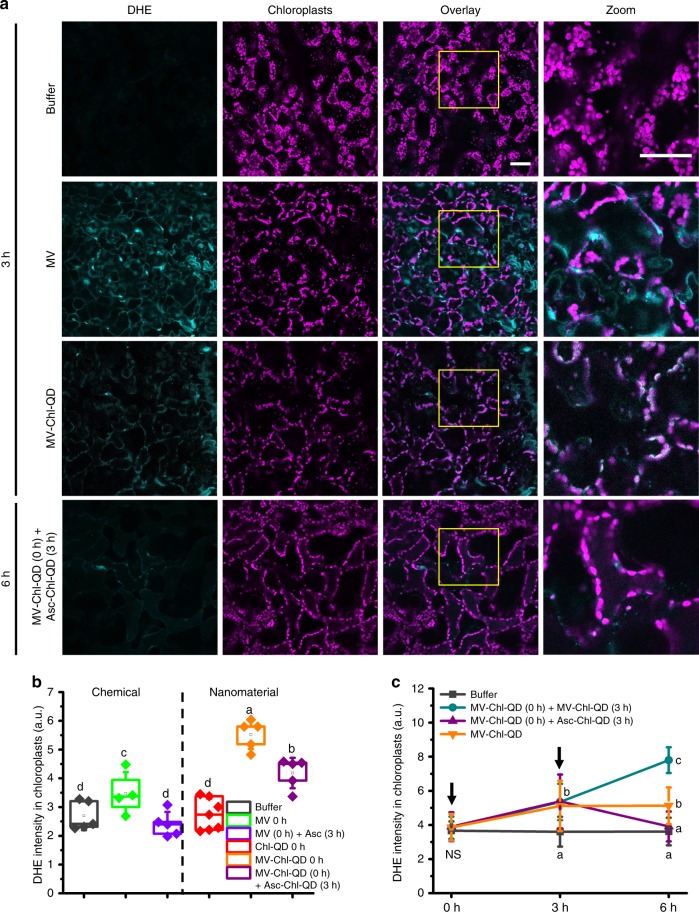
Chloroplast specific subcellular tuning of oxidative status by targeted delivery of nanoparticles with biochemical cargoes. **a** Confocal microscopy images of *Arabidopsis* leaf mesophyll cells illustrating the targeted generation and scavenging of superoxide anion (detected by DHE fluorescent dye) in chloroplasts by MV-Chl-QD and Asc-Chl-QD guided by peptide recognition motifs, respectively. Scale bar, 40 µm. **b** Comparison between chemical and nanotechnology-based approaches for specifically increasing superoxide in chloroplasts. DHE was used as an indicator for superoxide ROS levels after 6 h. Chemicals and nanomaterials were treated at the time points specified in the legend. Box plot error bars represent standard deviation, boxes are the interquartile range from the first to the third quartile with squares as the medians, and horizontal line represents the mean. **c** Temporal patterns of DHE fluorescence signal intensity inside chloroplasts in leaf mesophyll cells showing the specific increase and subsequent decrease of chloroplast superoxide anion levels in plants infiltrated with MV-Chl-QD (*n* = 4) at time 0 h and subsequently perfused with Asc-Chl-QD at time 3 h (purple line). A steady increase in DHE intensity was observed in leaves treated with MV-Chl-QD at time 0 h and 3 h (*n* = 5) (cyan line). Leaves infiltrated with MV-Chl-QD (*n* = 4) at time 0 h only (orange line) showed an increase in DHE signal that plateaus. Controls were performed for leaves infiltrated only with buffer (*n* = 5) (dark gray line). Values are means and ± indicates standard deviation. Statistical comparisons were performed by one-way ANOVA based on Duncan’s multiple range test (two tailed). Lower case letters represent significance at *P* < 0.05. NS denotes not significant.

Current methods of MV and Asc delivery in plants rely on foliar or soil absorption in aqueous solutions^45,48^. However, MV is a non-selective herbicide that reacts with NADPH in the apoplast, chloroplasts, mitochondria and peroxisome, thus non-specifically increasing both intra- and extra-cellular levels of superoxide anion^49,50^. Through confocal fluorescence microscopy in *Arabidopsis* leaf mesophyll cells, we measured the levels of ROS colocalization with chloroplasts and intensity changes using DHE (dihydroethidium) dye, a superoxide anion indicator (Fig. 6a). Leaves treated with MV alone exhibited superoxide anion signals both in chloroplasts and surrounding plant cell membranes and organelles. In contrast, most superoxide anion generation was detected in chloroplasts of leaves infiltrated with MV loaded Chl-QD (MV-Chl-QD). We observed a significantly higher degree of colocalization between chloroplasts and DHE induced by MV-Chl-QD (78.8 ± 7.0%) (± indicates standard deviation, *n* = 7) than by MV chemical alone (32.2 ± 11.2%) (± indicates standard deviation, *n* = 11) (*P* < 0.001) (Supplementary Fig. 7). In comparison with chemical application of MV (*n* = 4) alone or MV plus Asc (*n* = 10) without nanoparticles, the developed MV-Chl-QD (*n* = 10) and Asc-Chl-QD (*n* = 9) resulted in highly specific ROS manipulation within chloroplasts (Fig. 6b). Furthermore, the Chl-QD are able to tune the redox status of chloroplasts by MV-Chl-QD (*n* = 5) application that generates ROS in chloroplasts followed by Asc-Chl-QD (*n* = 5) that scavenges ROS in chloroplasts after 6 h (Fig. 6a, c). Control experiments, in which Chl-QD (*n* = 3) without cargoes were infused into leaves, resulted in no changes of DHE intensity in chloroplasts indicating that the nanoparticles alone are not responsible for the observed changes in ROS (Fig. 6b).

More than doubling the increase in targeted delivery of chemicals by nanomaterials, from 32.2% to 78.8%, could have a significant impact on minimizing agricultural inputs in the field and pollution of the environment. It may also allow studies in plant cell biology in wild-type and non-model plants that require specific organelle engineering. For example, ENM targeted delivery of biochemicals to subcellular compartments guided by peptide recognition motifs may improve our understanding of the role of signaling molecules (e.g., ROS) in plants. By specifically manipulating levels of ROS in chloroplasts we can gain insight into the role of this organelle in regulating redox sensitive physiological and developmental processes^51^. MV is a widely used herbicide, also named paraquat, that leads to toxic effects to mammalian cells due to its redox activity in mitochondria and has been linked to the development of Parkinson’s disease^52^. Our rational design of targeted biochemical delivery of ENM leveraging the plant molecular machinery could be utilized to create a variety of nanoparticle targeting approaches for nano-enabled agricultural applications.

## Discussion

Precise control of nanoparticle location in plants is crucial for the application of nanotechnology in plant biology, biotechnology, and nano-enabled agriculture. To date there are no approaches that leverage the plant molecular recognition machinery for controlling nanoparticle specific location and intended function in plant organelles. The use of guiding peptides provides a molecular tool for targeted delivery of nanomaterials to subcellular compartments with a precision not achieved before in plants. We demonstrated that by controlling QD size, zeta potential, and fluorescence emission range, we circumvent plant cell barriers for biochemical delivery allowing in vivo tracking with low interference from leaf background. Although Cd based QD properties make them ideal for detection by multiple advanced analytical tools including confocal fluorescence microscopy and elemental analysis by ICP-MS, chronic or high-level exposure of these nanoparticles can lead to toxic effects in organisms depending on their surface properties^53^. QD within the non-toxic exposure conditions of this study are useful model nanoparticle systems for basic research at the interface of plants and nanotechnology but applications in agriculture would require the use of environmentally friendly Cd-free QD^54^. However, the QD functionalization with a peptide recognition motif demonstrates an approach to engineer targeted nanomaterials for improved colocalization within organelles and delivery of biochemicals in vivo. The high localization efficiency and specificity of nanomaterials enabled by biorecognition surpasses that of conventional methods based on nanoparticle size and charge alone. As a result, the nanoparticle-mediated delivery of biochemicals such as MV and Asc to chloroplasts specifically allows tuning of their redox status while significantly minimizing ROS generation or scavenging in other subcellular compartments.

Although we demonstrate that biochemicals including MV and Asc are delivered to chloroplasts with high efficiency (78.8%), further research on modifying guiding peptide properties including spacer amino acids, hydrophilicity, and charge, among others, may allow enhancing the delivery specificity to plant organelles while avoiding unwanted exposure to other plant cell compartments. We expect that increasing the amino acid spacer length would be needed for nanoparticles coated with ligands larger than those used in this study (e.g., plasmid DNA, RNA). Tuning hydrophobicity of the peptide recognition motif is also likely to affect the nanoparticle translocation in plant cells as it was shown by previous studies coating nanoparticles with amphiphilic polymers^29^. Models of plant cell and chloroplast uptake based on nanoparticle size and charge alone using polymeric ligands^38,39^ do not explain why Chl-QD coated with biorecognition motifs with lower zeta potential magnitude than MPA-QD exhibit higher colocalization rates with chloroplasts. This indicates that nanoparticle-plant interaction models should incorporate engineered biomolecule coatings and acquired coronas for making accurate predictions of the distribution of nanoparticles in plants.

Our comparative analysis of the designed chloroplast guiding peptide with highly conserved sequence recognition motifs in Rubisco small subunits from other plant species, suggests that biorecognition motif targeted delivery approaches can be translated to a wide range of dicot plant species. This study highlights strategies to create nanobiotechnology tools that can bypass plant biological barriers for targeted delivery of biomolecules and chemical cargoes to chloroplasts for fundamental research in plant biology and more efficient delivery of agrochemicals to crops. The biorecognition approach of coating nanoparticles with guiding peptides for targeted delivery could be extended to other types of nanomaterials for applications including genetic element delivery, targeted delivery of sensors, nutrients or pesticides to specific plant tissues or subcellular compartments.

## Methods

### Plant growth

Plants were grown in Adaptis 1000 growth chambers (Conviron) using standard conditions^55^. Growth chamber conditions were 200 μmol m^−2^ s^−1^ PAR, 24 ± 1 and 21 ± 1 °C day/night, 60% humidity, and 14/10 h day/night regime. Plants were watered once every three days. Four-week-old Columbia ecotype (Col-0) *Arabidopsis thaliana* plants (seed stock source CS60000) were used for this study.

### Synthesis of quantum dots with chloroplast targeting peptide

The CdTe/CdS quantum dots were prepared by the reaction between CdCl_2_ and NaHTe solution in the presence of mercaptopropionic acid solution (MPA) as the stabilizing agent^56–59^. First, 0.01 g of CdCl_2_ and 50 μL of mercaptopropionic acid were dissolved in 50 mL of ddH_2_O forming a colloidal solution. The resulted Cd/MPA colloidal solution was adjusted to pH 11 with NaOH solution (0.1 M) and stirred for 15 min under reflux. Meanwhile, NaHTe solution was prepared by dissolving 0.05 g of NaBH4 and 0.02 g of tellurium powder in 0.6 mL of 50% ethanol in a 20 mL glass vial. NaHTe was allowed to react at 70 °C with gentle stirring for 5 min. The reaction vial was lightly capped to avoid excess oxygen from oxidizing the reactants. The NaBH_4_ and tellurium mixture exhibited a color change as the reaction progressed turning black-blue to pink-purple in color. Immediately after the color change, 150 µL of freshly prepared NaHTe was added to the Cd/MPA colloidal solution under reflux conditions. Following reflux, an increase in fluorescence of the solution could be monitored when excited under UV light. Aliquots of quantum dot solution were collected at 5 minutes. The emission of QD could be tuned to a specific wavelength by adjusting the reaction time. The resultant MPA-QD absorbance, size, zeta potential, and emission (under 405 nm excitation) were characterized accordingly. QD for targeted delivery of biochemicals to chloroplasts (Chl-QD) were designed and synthesized with size that facilitates translocation across leaf cell walls, fluorescence within the low background optical window for leaves, and coated with a truncated RbcS guiding peptide (GeneScript) to target chloroplasts as described in multiple steps outlined below and in Supplementary Fig. 1. RbcS peptide was randomized by using http://www.bioinformatics.org/sms2/shuffle_protein.html javascript suite^60^. All chemicals were purchased from Sigma Aldrich unless otherwise stated.

The preparation of p-aminophenyl boronic acid capped QD (APBA-QD) was performed as follows. The MPA-QD terminal carboxyl group was functionalized by 1-ethyl-3-(3-dimethylaminopropyl) carbodiimide (EDC) and N-hydroxysuccinimide (NHS) activated reaction^57,61^. Briefly, NHS (2000 nmol) and EDC/HCl (2000 nmol) was added to the 1 nmol of the MPA-capped QD in TES buffer (10 mM TES buffer, pH 7.4). Then, the mixture was gently stirred (500 rpm) for 15 min at room temperature. Next, 80 μl of a 25 mM APBA solution was added to the activated MPA-QD solution to generate aminophenyl boronic acid functionalized quantum dots (APBA-QD). The reaction was stirred (500 rpm) for 3 h at room temperature. Finally, the excess of APBA was removed by washing at least twice through a 10 K amicon filter with ddH_2_O. The APBA-QD solution was sonicated for 30 min at 80% power at 37 kHz to break down any agglomerated particles.

For synthesis of β-CD-capped QD, the APBA-QD were dissolved in 10 mM TES buffer (pH 10.4). Then 1 μmol of mono-(6-ethanediamine-6-deoxy)-β-cyclodextrin (β-CD, Cavcon) in water was added to the APBA-QD solution and the resulting mixture was reacted overnight at room temperature with gentle stirring (500 rpm)^61^. The excess of β-CD was removed by washing with a 10k Amicon filter followed by sonication for 30 min at 80% power at 37 kHz. The resulting β-CD coated quantum dots (CD-QD) were suspended in 10 mM TES (pH 7.5).

For peptide-conjugated β-CD-capped QD preparation, 1 μmol NHS-PEG_4_-MAL linker (succinimidyl-[(N-maleimidopropionamido)-tetraethyleneglycol] ester, Thermo Fisher Scientific, USA) was added to the surface of CD-QD by reacting with its terminal amine to form a covalent bond^57,61^. The mixture was incubated at ambient temperature for 1 h with gentle stirring (500 rpm). The excess NHS-PEG_4_-MAL was removed by washing the mixture through a 10 K Amicon column with ddH_2_O and the product was suspended in 10 mM TES (pH 8.0). Finally, 1 μmol of RbcS chloroplast targeting peptide was added to MAL-PEG_4_-QD and allowed to react for 1 h at room temperature with gentle stirring (500 rpm). The RbcS peptide dissolved in DMSO was diluted with TES buffer to adjust the pH to 8.0. The resulting chloroplast targeting quantum dot (Chl-QD) was pulse centrifuged for 30 seconds at 3500 rpm to remove large agglomerates of non-conjugated peptide. The Chl-QD can be stored up to one week without significant aggregation.

### Methyl viologen and ascorbic acid loading to quantum dots

Loading of methyl viologen and ascorbic acid into β-CD conjugated onto Chl-QD was carried out by adding MV and Asc in excess (0.1 mM) to an aqueous solution of 200 nM (0.17 mg mL^−1^) Chl-QD in 10 mM TES buffer pH 7.0. The mixture of MV-Chl-QD or Asc-Chl-QD was vortexed and incubated for 0.5 h, and washed once through an Amicon 10 K filter with ddH_2_O to remove excess molecules. MV and Asc exhibit the maximum absorbance at 260 and 265.5 nm, respectively. The inclusion complex concentration (MV-Chl-QD and Asc-Chl-QD) was calculated based on the absorbance at 260 or 265.5 nm of reference to unloaded Chl-QD^36,62^. The resultant MV-Chl-QD or Asc-Chl-QD concentration was extrapolated using a standard curve (Supplementary Fig. 2b–d). The final dosage of chemicals infiltrated into plants with 200 nM of Chl-QD was 60 µM MV or 60 µM Asc in 100 µL TES buffer (pH 7.0) (Supplementary Fig. 2c, d). To compare with chemicals alone, the same concentration of chemicals was applied to plants, 60 µM MV or 60 µM Asc in 100 µL TES buffer (pH 7.0).

### Isothermal titration calorimetry (ITC)

ITC of cyclodextrin functionalized QD (CD-QD) or MPA-QD with chemical cargoes (Asc or MV) was performed using a MicroCal iTC200 instrument (GE Healthcare). QD and Asc or MV were dissolved in 10 mM TES buffer, pH 7.3 at 25 °C. The concentrations of QD were set at 0.5 μM and the concentration of injected Asc and MV was 25 mM. The volume of each injection was 2 μL and a total of 21 injections were performed at 180 s intervals with a reference power of 5 μcal s^−1^. The ITC curves were analyzed with Origin (MicroCal) using a one-set-of-sites fitting model. The bound fractions of Asc and MV on QD in the final solution (with initial Asc or MV loading of 60 μM) injected into leaves was calculated based on the following equation^63^:

$$\left[ {A_{{\mathrm{bound}}}} \right] \;=\; \frac{{n[QD]_0[A]}}{{K_d \;+ \;[A]}}$$

where [*A*_bound_] and [*A*] are the concentration of bound and unbound chemicals in solution, respectively, *n* is the number of binding sites on QD, [*QD*]_0_ is the initial QD concentration, and *K*_*d*_ is the dissociation constant between QD and chemicals.

### Nanomaterial characterization

All nanomaterials were characterized for their absorbance in the UV-vis, hydrodynamic size, zeta potential, and fluorescence emission. Surface functional groups were analyzed by FTIR. Zeta potential and hydrodynamic sizes of nanomaterials were measured in DI water (pH 7) using a Malvern Zetasizer (Nano ZS) and sizer (Nano S), respectively. UV-vis absorption spectra were collected using a UV-2600 Shimadzu spectrophotometer. The sample was prepared in a quartz cuvette filled with 1 mL of a 1:10 fold dilution of nanoparticles. The concentration of the nanomaterials (mol L^−1^) was determined using Lambert-Beer’s law (Eq. 1) where Abs is absorbance, ${\it{\epsilon }}$ is the extinction coefficient, L is the path length, and c is concentration. Equation 2 refers to the extinction coefficient (${\it{\epsilon }}$), calculated based on the QD hydrodynamic diameter (d)^64^. The QD absorbance at 465 nm was used to determine the QD concentration in solution (Eq. 1).

1 $${\mathrm{Abs}} \;= \;{\it{\epsilon }} \times L \times c,$$

2 $${\it{\epsilon }} \;=\; 10043 \times ({\mathrm{d}})^{2.12}$$

Transmission electron microscopy (TEM) was performed on a Philips FEI Tecnai 12 microscope operated at an accelerating voltage of 120 kV. The TEM samples were prepared by placing one drop of particle solution (0.5 µM) onto the grid (ultrathin carbon film on lacey carbon support film, 400 mesh, Cu, Ted Pella) followed by drying naturally. The surface coatings and functional groups on nanomaterials were characterized by Fourier transform infrared spectroscopy (FTIR) using a Bruker spectrometer (Alpha I). Samples from each step in the synthesis of Chl-QD were taken to analyze functional groups on the nanoparticle surface (Fig. 2e).

### Nanoparticle delivery into plant leaves

All nanoparticles infused through the *Arabidopsis* leaf lamina were suspended in 10 mM TES buffer (pH 7.0). The Chl-QD solution was diluted to 200 nM (0.17 mg mL^−1^) and loaded with 60 µM methyl viologen or ascorbic acid. Nanoparticle solution was infused through the abaxial side of the leaf using a 1 mL needles syringe^55,65^. Approximately 100 μL solution was perfused into each plant leaf by gently pressing the tip of the syringe against the bottom of the leaf lamina and depressing the plunger. The excess solution was gently removed from the leaf surface by kimwipes.

### Confocal fluorescence microscopy imaging

*Arabidopsis* leaf samples were imaged by a Leica laser scanning confocal microscope TCS SP5 (Leica Microsystems, Germany)^26,66^. Each leaf was infused with 200 nM (0.17 mg mL^−1^) Chl-QD, MV-Chl-QD or Asc-Chl-QD and incubated for 3 h. After incubation, a leaf punch was excised and incubated in 10 µM DHE (Thermo Fisher Scientific, USA) in 10 mM TES buffer (pH 7.0) for 30 min. The leaf was immediately placed on a glass slide equipped with Carolina observation gel for confocal analysis^63^. A pea-size amount of observation gel (Carolina) was placed on a glass slide and pressed to about 1 mm thin on slides. A cork borer was used to cut a circular section of gel roughly twice the size of the leaf discs at the center of the observation gel and a leaf disc was placed in the within cavity. The imaging settings were as follows: ×40 wet objective (Leica Microsystems, Germany); 405 nm laser excitation for QD; 514 nm for DHE; z-stack section thickness = 2 µm; line average = 4. The PMT detection range was set 500–550 nm for QD; 580−615 nm for DHE; and 720−780 nm for chloroplast autofluorescence. The confocal imaging of QD and DHE signals were conducted separately to avoid the overlap between excitation of DHE dye and emission detection range of QD. Three to eight individuals (4 leaf discs for each plant) in total were used. The z-stacks (“xyz”) of two different regions were taken per leaf disc.

All confocal microscopy images were analyzed using FIJI (ImageJ) in which QD, DHE, and chloroplast images were evenly divided by drawing six lines of the region of interest (ROI), with the same length and distance between each ROI line^26,40^. The corresponding fluorescence intensity profiles of QD and DHE fluorescence and chloroplast autofluorescence were then measured across the six ROI line sections and reported as a subset of the image showing signal intensity plot. The percentage of chloroplasts colocalized with QD was counted as the overlapped peaks of fluorescence emission of chloroplast pigments and QD or DHE fluorescence signals. For DHE intensity analysis in chloroplasts, the pixel intensity of DHE fluorescence was measured (FIJI) in a ROI enclosing chloroplasts and reported as mean DHE intensity.

### Chloroplast isolation

Chloroplasts were isolated through a centrifugation gradient method^17,67^. Intact chloroplasts were isolated from plants treated with 500 nM of Chl-QD, or buffer (10 mM TES pH 7.3). Approximately 100 µL of the solution was infiltrated into primary leaf whorl of 3–4-week-old *Arabidopsis thaliana* plants. Approximately 8 g of leaf tissue with or without nanoparticles was collected from 5–6 plants per treatment. Leaf tissue was macerated in 1X chilled sucrose buffer (pH 7.3, 28 mM Na_2_HPO_4_, 22 mM KH_2_PO_4_, 2.5 mM MgCl_2_, 400 mM sucrose, and 10 mM KCl) by two cycles of centrifugation at 4000 RCF for 10 min. Immediately following isolation, a sample of intact chloroplasts was placed on a glass slide for detection of quantum dot fluorescence within extracted chloroplasts using confocal microscopy.

### Inductively coupled plasma mass spectrometry (ICP-MS)

Following chloroplast isolation, sample pellets (~0.1 g) were air-dried for 48 h, placed in 50 mL polypropylene digestion tubes, and digested with a solution of 5% HNO_3_/1% HCl/1% H_2_O_2_ v/v. Samples were first digested in 1 mL of HNO_3_/ 0.4 mL of HCl and heated at 115 °C for 5 min using a hot block (DigiPREP System; SCP Science, Champlain, NY). Then, 0.4 mL of H_2_O_2_ was added and incubated for an additional 10 min. The solution was further diluted and analyzed by ICP-MS (Agilent 7700x ICP-MS) to quantify the content of Cd and Te. Individual element concentrations were calculated in μg g^−1^ (element mass in μg per gram of chloroplast dry mass).

### Statistical analysis

All data were represented as mean, ± indicates standard deviation (SD), and *n* = biological replicates or independent nanoparticle sample replicates. Statistical analysis was performed using SPSS 23.0. Statistical comparisons were performed by one-way ANOVA based on Duncan’s multiple range test (two tailed) or independent samples *t*-test (two tailed). All data were subjected to normal distribution tests by using non-parametric tests based on 1-Sample K-S (Kolmogorov-Smirnov test).

## Supplementary information

Supplementary Information

Description of Additional Supplementary Files

Supplementary Movie 1

Source Data File

## Data Availability

The data underlying Figs. 2a–e, 3b, 4b, 5a, b, and 6b, c, as well as Supplementary Figs. 2a–d, 5c, 6a, b and 7 are provided as a source data file. Any other data supporting the findings of this study are available in the manuscript and its supplementary files or from the corresponding author upon request.
