# Supplementary material for: Targeted delivery of nanomaterials with chemical cargoes in plants enabled by a biorecognition motif
Source: Nat Commun. 2020 Apr 27;11:2045. doi: 10.1038/s41467-020-15731-w (PMC7184762; doi:10.1038/s41467-020-15731-w)
Supplement: Supplementary file 1 — Supplementary Information [file 41467_2020_15731_MOESM1_ESM.pdf]

Supplementary Information

Targeted Delivery of Nanomaterials with Chemical Cargoes in Plants Enabled by a Biorecognition Motif

Santana et al.

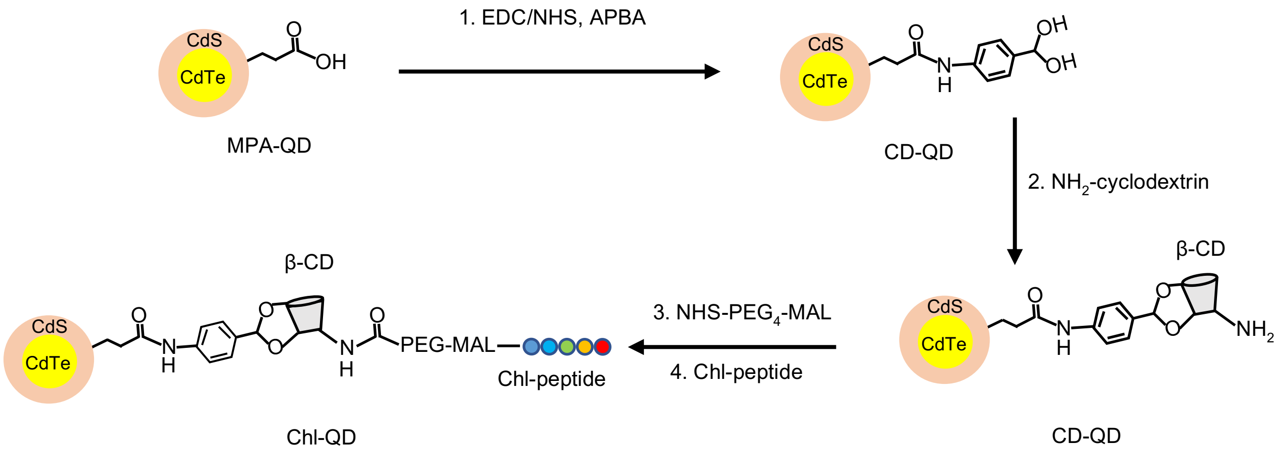

Supplementary Figure 1 | Schematic diagram of stepwise synthesis of targeted nanomaterials with biorecognition motifs. Diagram illustrates step by step synthesis of chloroplast targeting quantum dots (Chl-QD) containing β-cyclodextrin (β-CD) molecular baskets and chloroplast guiding peptides. The targeting peptide design (Chl-peptide) is based on a truncated Rubisco small subunit biorecognition motif (RbcS) that guides protein precursors to chloroplast outer membranes.

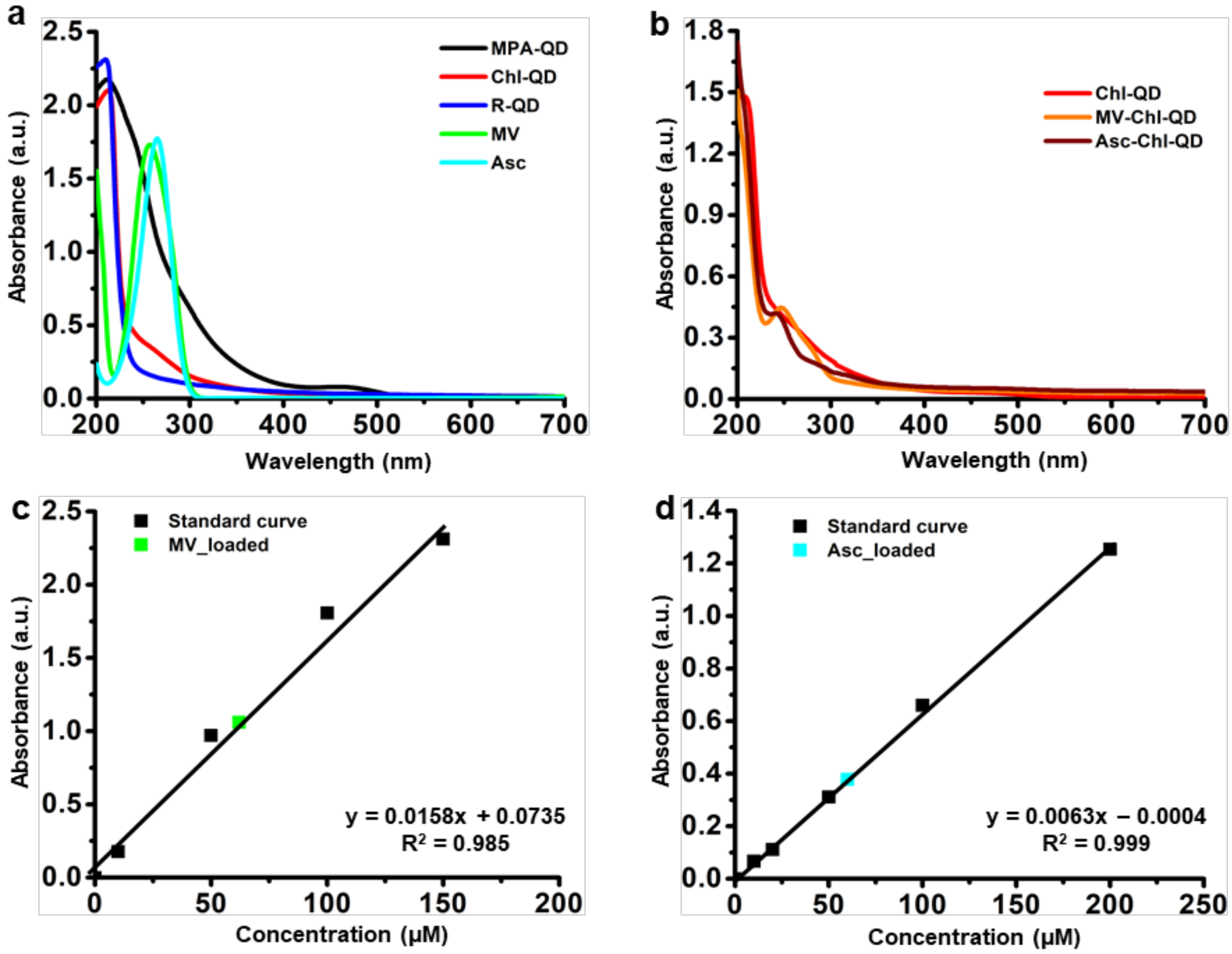

Supplementary Figure 2 | Absorption spectra of Chl-QD and loading efficiency of MV and Asc in Chl-QD. a, UV-vis absorption spectra of QDs coated with MPA (MPA-QD), targeting peptide (Chl-QD) and random peptide (R-QD), MV (methyl viologen) and Asc. (ascorbic acid). **b,** Absorbance spectra of Chl-QD before and after loading with MV (MV-Chl-QD) and Asc (Asc-Chl-QD). Calibration curves of **c,** MV and **d,** Asc absorbance versus concentration were used to determine the loading efficiency of MV (green square) and Asc (cyan square) in Chl-QD.

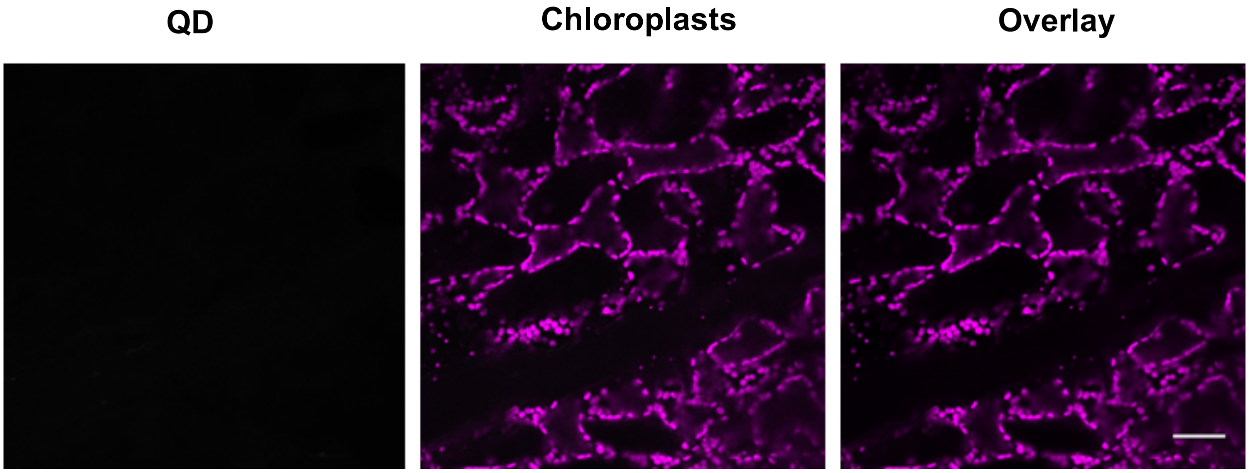

Supplementary Figure 3 | Confocal microscopy images of Arabidopsis leaf mesophyll cells infiltrated with TES buffer as control. Leaves infiltrated with TES buffer exhibit no fluorescence signal for QDs within the detection range for QD emission (500 -550 nm). Scale bar, 50 μm.

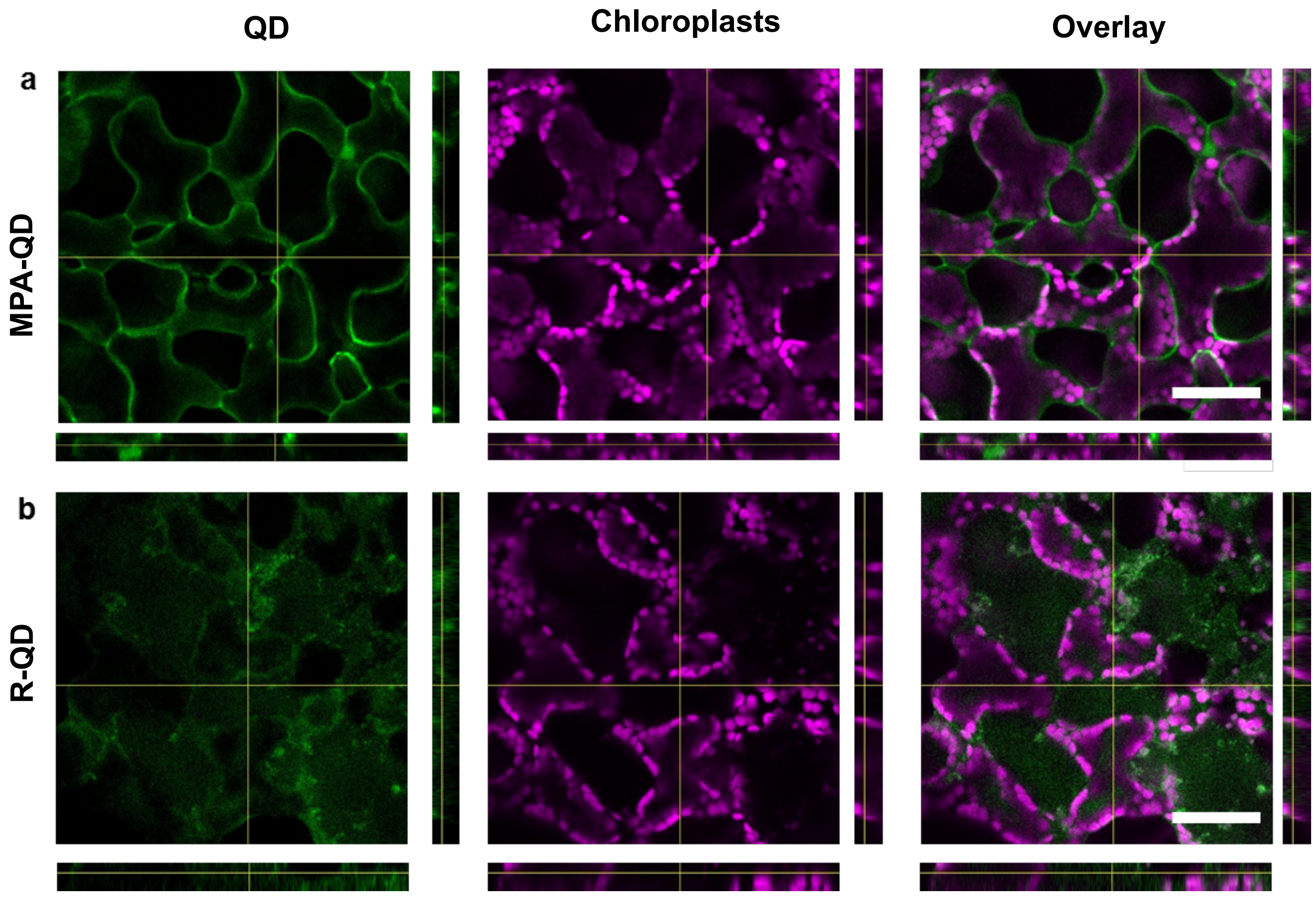

Supplementary Figure 4 | Orthogonal views of confocal microscopy images between QD and chloroplasts. Projections in the z-axis of confocal microscopy images in the x and y planes showing colocalization of nanoparticles with chloroplasts for **a,** QDs coated with MPA (MPA-QD) and **b,** random peptide (R-QD). Z axis optical slices were taken every 2 μm up to a depth of 20 μm. Scale bar 50 μm.

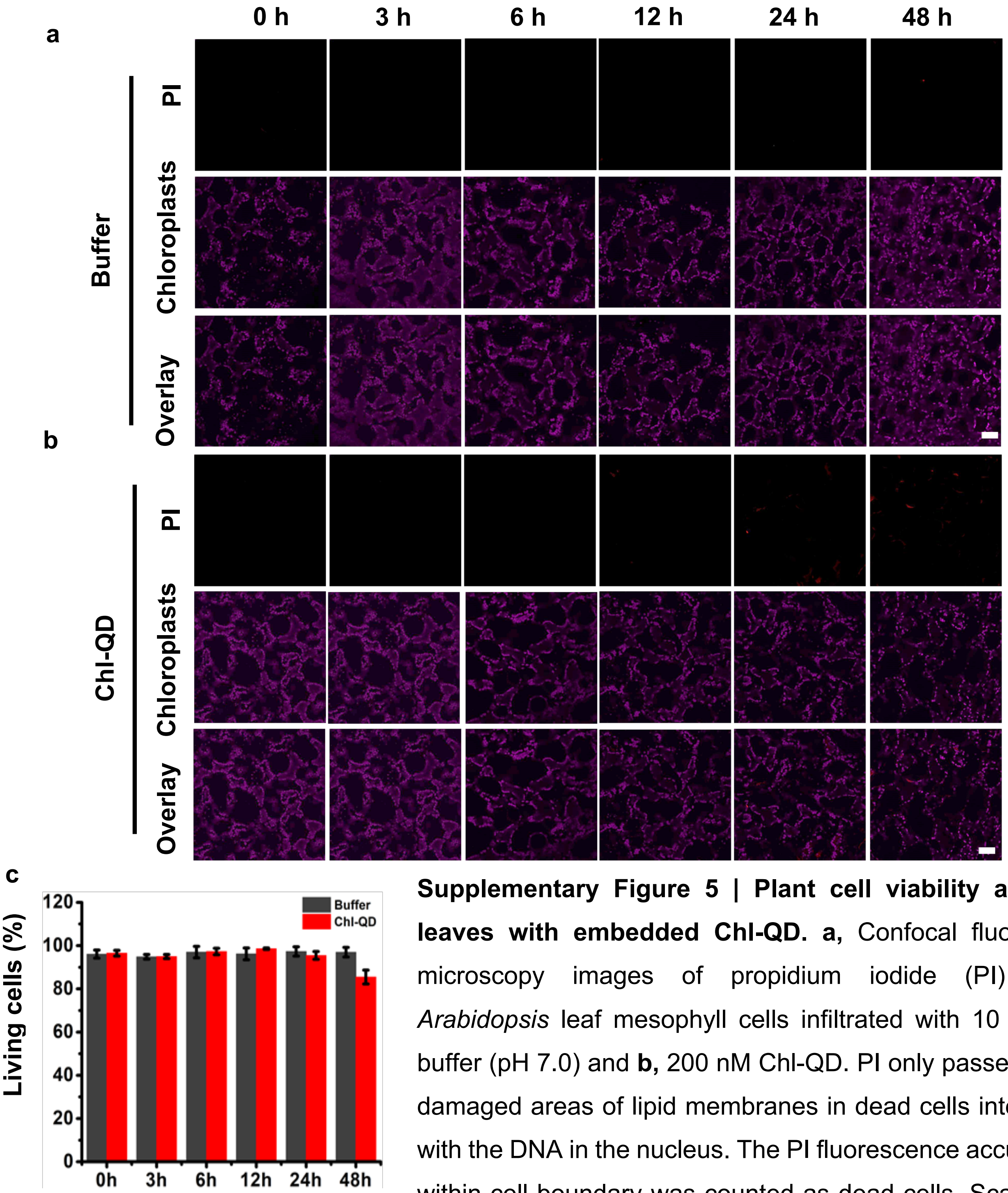

Supplementary Figure 5 | Plant cell viability assays in leaves with embedded Chl-QD. a, Confocal fluorescence microscopy images of propidium iodide (PI) stained *Arabidopsis* leaf mesophyll cells infiltrated with 10 mM TES buffer (pH 7.0) and **b,** 200 nM Chl-QD. PI only passes through damaged areas of lipid membranes in dead cells intercalating with the DNA in the nucleus. The PI fluorescence accumulation within cell boundary was counted as dead cells. Scale bar 50 µm. **c**, The percentage of living cells in Arabidopsis leaves infiltrated with Chl-QD or TES buffer solution. Mean ± SD, *n* = 4. Error bars represent standard deviations.

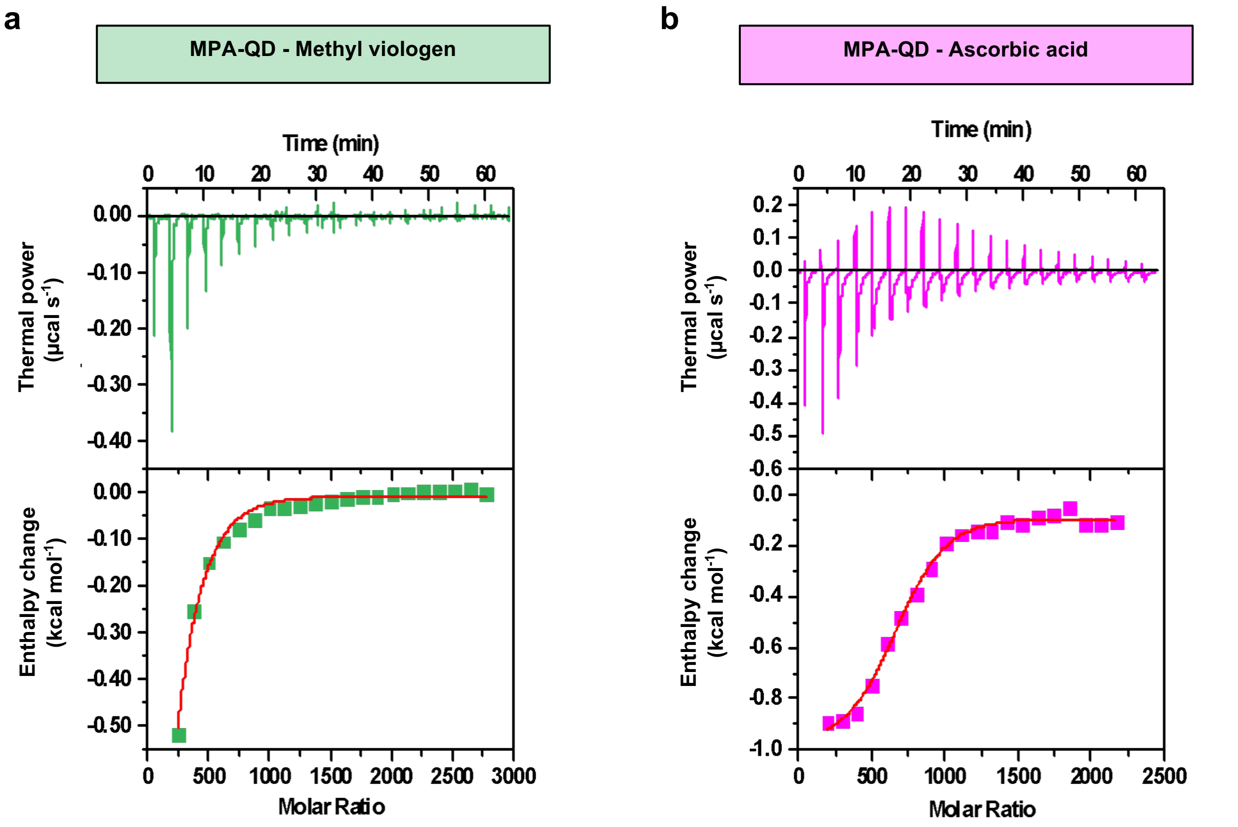

Supplementary Figure 6 | Isothermal titration calorimetry of 3-Mercaptopropionic acid (MPA) coated quantum dots (MPA-QD) with chemical cargoes. Thermograms (top) and binding isotherms (bottom) of MPA-QD interacted with **a**, methyl viologen and **b**, ascorbic acid.

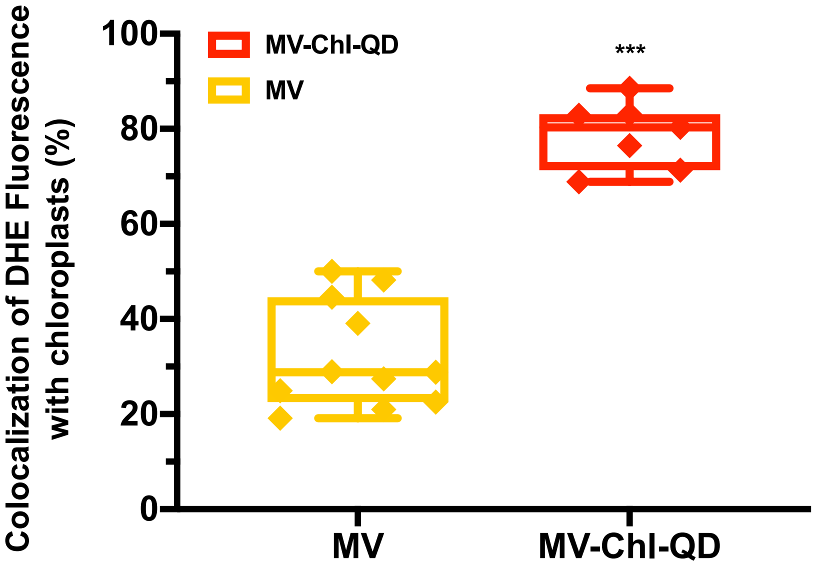

Supplementary Figure 7 | Comparison of DHE fluorescence localized within chloroplasts in leaves infused with MV chemical and MV-Chl-QD. *Arabidopsis* leaves treated with Chl-QD loaded with MV and targeted to chloroplasts have significantly higher colocalization rates of DHE (fluorescent dye for superoxide anion) with chloroplasts (78.8 ± 7.0%, Mean ± SD, *n* = 7) than leaves treated with MV chemical alone (32.2 ± 11.2 %, Mean ± SD, *n* = 11). DHE fluorescence intensities were measured after a 3 h incubation with Chl-QD and MV treated leaves. Error bars represent standard deviation and boxes represent the interquartile range from the first to the third quartile with squares as the medians and horizontal line with representative treatment color represents mean. Box plots contain diamond symbols for each data point. Statistical comparison was performed by independent samples t-test (two tailed). *** indicates P < 0.001.

**Supplementary Table 1: Wide range of chemicals forming inclusion complexes with cyclodextrins.**		
**Chemicals**	**Function**	**Reference**
Allyl isothiocyanate	Antimicrobial	Li et al. 2007
Chlorpyrifos	Insecticide	Lucas-Abellán et al. 2008
Hesperidin, naringenin, naringin	Flavonoids	Ficarra et al. 2002
2-methyl-5-(1-methylethyl) (carvacrol)	Reduce intestinal parasitic infection	Shashank et al. 2018
Nicotinic acid, ascorbic acid	Vitamins and antioxidants, Plant metabolite	Garnero et al 2007, Palomar-Pardavé et al. 2011, Subhadeep et al. 2016,
Methyl viologen	Herbicide	Sivagnanam et al 1992, Ong et al 2003, Mondal et al. 2016
Dihydroxyphenylalanine	Neurotrophic factor	Palomar-Pardavé et al. 2011
Theophylline	Alkaloid treatment of respiratory diseases	Amarr et al. 1996
Amatadine	Antiviral agent	Ai et al. 2012
Beta-Carotene, carotenoids	Vitamin, plant metabolite	Kaur et al. 2016,

Nitrophenol isomers	Pharmaceutical precursor	Zhang et al. 2015
Alkaline phosphatase	Clinical disease indicator	Jia et al. 2010
Napthalene	Insecticide	Harata et al. 1975
Terfenadine	Antihistamine	Choi et al. 2001
Carvedilol	Antioxidant	Wen et al. 2004
Sulindac, Fenoprofen	Non estradiol anti-inflammatory	Diaz et al. 1999
Albendazole	Intestinal parasite treatment	Garcia et al. 2014
Cocaine	Stimulant	Nesnas et al. 2000
MCPA 4-chloro-2-methylphenoxyacetic acid	Herbicide	Garrido et al. 2014
Norflurazon	Herbicide	Villaverde et al. 2004

Supplementary References

Ai, X., Niu, L., Li, Y., Yang, F. & Su, X. A novel β-Cyclodextrin-QDs optical biosensor for the determination of amantadine and its application in cell imaging. Talanta 99, 409–414 (2012).

Ammar, H. O., Ghorab, M., El-nahhas, S. A., Omar, S. M. & Ghorab, M. M. Improvement of some pharmaceutical properties of drugs by cyclodextrin complexation. 5. Theophylline. *Pharmazie* **51,** 42–46 (1996).

Choi, H.-G. et al. Terfenadine–β-Cyclodextrin Inclusion Complex with Antihistaminic Activity Enhancement. Drug Dev. Ind. Pharm. 27, 857–862 (2001).

de Oliveira, V. E. et al. Carotenoids and β-cyclodextrin inclusion complexes: Raman spectroscopy and theoretical investigation. J. Phys. Chem. A 115, 8511–8519 (2011).

Díaz, D., Escobar Llanos, C. M. & Bernad Bernad, M. J. Study of the binding in an aqueous medium of inclusion complexes of several cyclodextrins involving fenoprofen calcium. Drug Dev. Ind. Pharm. 25, 107–110 (1999).

Ficarra, R. et al. Study of flavonoids/beta-cyclodextrins inclusion complexes by NMR, FT-IR, DSC, X-ray investigation. J. Pharm. Biomed. Anal. 29, 1005–1014 (2002).

Garrido, J., Cagide, F., Melle-Franco, M., Borges, F., Garrido, E.M. Microencapsulation of herbicide MCPA with native β-cyclodextrin and its methyl and hydroxypropyl derivatives: An experimental and theoretical investigation. J. Mol. Struct. 1061, 76–81 (2014).

García, A., Leonardi, D., Vasconi, M. D., Hinrichsen, L. I. & Lamas, M. C. Characterization of albendazole-randomly methylated-β-cyclodextrin inclusion complex and in vivo evaluation of its antihelmitic activity in a murine model of Trichinellosis. PLoS One 9, e113296 (2014).

Garnero, C. & Longhi, M. Study of ascorbic acid interaction with hydroxypropyl-beta-cyclodextrin and triethanolamine, separately and in combination. J. Pharm. Biomed. Anal. 45, 536–545 (2007).

Harata, K. & Uedaira, H. The Circular Dichroism Spectra of the β-Cyclodextrin Complex with Naphthalene Derivatives. BCSJ 48, 375–378 (1975).

Kaur, M., Bawa, M. & Singh, M. Β–carotene-β–cyclodextrin inclusion complex: towards enhanced aqueous solubility. Plan. Perspect. 5, 2016

Jia, L. et al. Fluorescence detection of alkaline phosphatase activity with β-cyclodextrin-modified quantum dots. Chem. Commun. 46, 7166–7168 (2010).

Lucas-Abellán, C., Gabaldón-Hernández, J. A., Penalva, J., Fortea, M. I. & Núñez-Delicado, E. Preparation and characterization of the inclusion complex of chlorpyrifos

in cyclodextrins to improve insecticide formulations. J. Agric. Food Chem. 56, 8081–8085 (2008).

Li, X., Jin, Z. & Wang, J. Complexation of allyl isothiocyanate by α- and β-cyclodextrin and its controlled release characteristics. Food Chem. 103, 461–466 (2007).

Mondal, S. & Purkayastha, P. α-Cyclodextrin Functionalized Carbon Dots: Pronounced Photoinduced Electron Transfer by Aggregated Nanostructures. J. Phys. Chem. C 120, 14365–14371 (2016).

Nesna, N., Lou, J. & Breslow, R. The binding of cocaine to cyclodextrins. Bioorg. Med. Chem. Lett. 10, 1931–1933 (2000).

Ong, W. & Kaifer, A. E. Salt effects on the apparent stability of the cucurbit [7] uril- methyl viologen inclusion complex. J. Org. Chem. 69, 1383–1385 (2004).

Palomar-Pardavé, M. et al. Electrochemical and spectrophotometric determination of the formation constants of the ascorbic acid-β-cyclodextrin and dopamine-β-cyclodextrin inclusion complexes. J. Incl. Phenom. Macrocycl. Chem. 69, 91–99 (2011).

Sivagnanam, U. & Palaniandavar, M. Selective inclusion of methyl viologen by β-cyclodextrin: effect of cyclodextrins on the electrochemistry of methyl viologen. J. Electroanal. Chem. 341, 197–207 (1992).

Tros de Ilarduya, M. C., Martín, C., Goñi, M. M. & Martínez-Ohárriz, M. C. Solubilization and interaction of sulindac with beta-cyclodextrin in the solid state and in aqueous solution. Drug Dev. Ind. Pharm. 24, 301–306 (1998).

Villaverde, J., Morillo, E., Perez-Martinez, J.I., Gines, J.M., Maqueda, C. Preparation and characterization of inclusion complex of norflurazon and β-cyclodextrin to improve herbicide formulations. J. Agric. Food Chem. 52, 864–869 (2004).

Wen, X., Tan, F., Jing, Z. & Liu, Z. Preparation and study the 1: 2 inclusion complex of carvedilol with β-cyclodextrin. J. Pharm. Biomed. Anal. 34, 517–523 (2004).

Zhang, Z., Zhou, J., Liu, Y., Tang, J. & Tang, W. Cyclodextrin capped CdTe quantum dots as versatile fluorescence sensors for nitrophenol isomers. Nanoscale 7, 19540–19546 (2015).
