# Supplementary material for: Targeted delivery of nanomaterials with chemical cargoes in plants enabled by a biorecognition motif
Source: Nat Commun. 2020 Apr 27;11:2045. doi: 10.1038/s41467-020-15731-w (PMC7184762; doi:10.1038/s41467-020-15731-w)
Supplement: Supplementary file 2 — Description of Additional Supplementary Files [file 41467_2020_15731_MOESM2_ESM.docx]

**Description of Additional Supplementary Files**

**File name:** Supplementary Movie 1

**Description:** Targeted delivery of quantum dots (green) to Arabidopsis chloroplasts (magenta) in leaf mesophyll cells is enabled by a guiding peptide recognition motif. A high level of colocalization between nanoparticles and chloroplasts is observed in the white overlay.
